# Expression, Purification, and Functional Analysis of Novel RelE Operon from *X. nematophila*

**DOI:** 10.1155/2014/428159

**Published:** 2014-11-27

**Authors:** Jitendra Singh Rathore, Lalit Kumar Gautam

**Affiliations:** ^1^Department of Microbiology, Perelman School of Medicine, University of Pennsylvania, 303 Johnson Pavilion, 3610 Hamilton Walk, Philadelphia, PA 19104, USA; ^2^School of Biotechnology, Gautam Buddha University, Greater Noida, Yamuna Expressway, Uttar Pradesh 201312, India

## Abstract

Bacterial toxin-antitoxin (TA) complexes induce programmed cell death and also function to relieve cell from stress by various response mechanisms. *Escherichia coli* RelB-RelE TA complex consists of a RelE toxin functionally counteracted by RelB antitoxin. In the present study, a novel homolog of RelE toxin designated as Xn-relE toxin from *Xenorhabdus nematophila* possessing its own antitoxin designated as Xn-relEAT has been identified. Expression and purification of recombinant proteins under native conditions with GST and Ni-NTA chromatography prove the existence of novel TA module. The expression of recombinant Xn-relE under tightly regulated ara promoter in *E. coli* Top 10 cells confirms its toxic nature in endogenous toxicity assay. The neutralization activity in endogenous toxicity assay by Xn-relEAT antitoxin confirms its antidote nature when studying the whole TA operon under ara regulated promoter. This study promotes newly discovered TA module to be regarded as important as other proteins of type II toxin-antitoxin system.

## 1. Introduction

In bacterial and archaeal genome toxin-antitoxin (TA) complex is evident [1]. Toxin-antitoxin modules are known to play role in plasmid maintenance and confer stability to them [2]. Bacterial toxin-antitoxin (TA) complex mainly comprises of a potent and stable toxin and its antidote labile antitoxin molecule. As understanding on toxin-antitoxin progresses, the role of these modules is found to provide protection to host from various stresses and manage to circumvent the problem by programmed cell death of the population [1]. They are said to act by prohibiting DNA invasion. Some physiological factors set the toxins free from their cognate antitoxin complex to keep the functionality of the cell unharmed. When bacterium comes under stress toxin gene is overexpressed and the produced toxin protein gets activated to interfere with cellular targets, while its cognate antitoxin protein is truncated by various means like Lon and Clp protease mediated degradation [3, 4]. There are three major classes of toxin-antitoxin (TA) module; among these, TA modules of class II form toxin-antitoxin protein complexes. Each antitoxin protein is capable of neutralizing the toxin encoded by the same TA module. TA modules are studied to be involved in bacterial persistence, drug tolerance, and multidrug resistance [5].

Toxin-antitoxin loci are extensively studied in* E. coli* where toxin RelE from RelBE module cleaves mRNA of a protein coding gene in a specific manner. Throughout the prokaryotic domain of living organisms, RelE toxins have widely conserved target sites, specificity, and functionality. To regulate the translation of specific gene RelE toxin competes with release factors and enters the site, where it can act on the mRNA [6, 7]. When cells are relieved from stress antitoxin protein replenishes its cognate toxin molecule, leading to resumption of growth following start of protein translation [6]. The amount of free toxin is found very less in the cell as its level is detrimental for the survival of the cell. Toxin concentration inside the cell is kept low by negative regulation of its transcription and through TA complex formation as well [8]. In nutrient stringent and other stress conditions RelB : RelE ratio is maintained via transcriptional regulation [9, 10]. Toxins are least susceptible to protease degradation whereas antitoxin molecules are degraded preferentially in that way. RelE toxin primarily acts as an endonuclease or it interferes with protein translation but also diminishes the synthesis of bacterial cell wall through various intermediate players [11, 12]. Either toxicity of the toxin molecules in a cell is normally neutralized with the cognate antitoxin by transcriptional repression of TA operon through binding to its palindromic sequences within its promoter region or it forms TA complex and resists toxin from binding to its target; all this happens in a manner defined as condition cooperativity [13–15].

*Xenorhabdus nematophila* is gram-negative, motile bacteria from Enterobacteriaceae family [16]. It establishes symbiotic relation with soil nematode from Steinernematidaefamily [17]. The bacteria help the nematode in killing the insect host, which is required to complete the life cycle of the nematode [16, 18]. During evolution few factors are likely conserved that help them to occupy their host by these pathogenic bacteria [19]. Earlier we have predicted the presence of three putative TA systems including* RelE* homolog in the genome of* X. nematophila* [20]. In this study we had emphasized the* RelE* homolog which acts as putative TA operon having toxin and antitoxin gene on its own. Encoded toxin protein is named as Xn-relE toxin, whereas its antidote is designed as Xn-relEAT.* Xn-relE* and* Xn-relEAT* genes from the TA modules were cloned and expressed in pGEXT41 and pET-28 expression system, respectively. After successful expression of the recombinant proteins, both toxin and antitoxin were purified by affinity chromatography using GST and Ni-NTA column under native conditions. Xn-relE gene alone and complete operon (*Xn-relE* +* Xn-relEAT*) were cloned under tightly regulated ara (arabinose regulated) promoter in pBAD vector. Endogenous toxicity assay with the pBAD constructs was performed in* E. coli* Top 10 cells by induction with arabinose.

## 2. Materials and Methods 

### 2.1. Bacterial Strain, Media, and Culture Conditions

All the chemicals and antibiotics were purchased from Sigma (Sigma-Aldrich) and HiMedia laboratories. Ligase, restriction endonucleases, and* Taq* polymerase were purchased from New England Biolabs (NEB), GST agarose resin was from Gold bioscience, USA, and Ni-NTA agarose resin and QIAquick spin columns were from Qiagen, Germany. Oligonucleotides were custom-synthesized by Imperial Life Sciences (ILS).* E. coli* strains DH5*α* (Bethesda Research Laboratories) were used as the host for cloning.* E. coli* BL 21(DE3) pLysS strain purchased from Novagen and* E. coli* Top 10 cells from Invitrogen were used in the endogenous assay. Chemical and salts used for protein purification and LB medium used for growing bacterial strains were purchased from HiMedia. Ampicillin, Kanamycin, and Chloramphenicol were used in the concentration of 100, 35, and 25 *μ*g mL^−1^, respectively.

### 2.2. Cloning in Expression Vector

**Figure 1:**
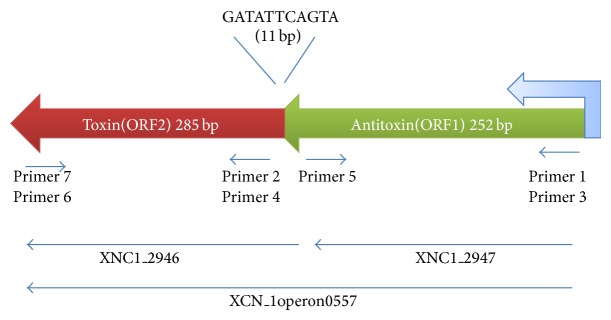
Genetic organization of toxin-antitoxin (TA) module of* Xn-relE* operon.* Xn-relE TA *operon is located on complementary strand in the genome of* X. nematophila*. Primers position and orientation for cloning of different domains depicted with arrows. Overlapping sequence of 11 nucleotide base pair is shown between the two modules. For cloning ORF 2 (285 bp) encoding Xn-relE toxin gene in pGEX4T expression vector, primer pairs 4 and 6 with* BamH*I site at 5′ end and* Xho*I site at 3′ end, respectively, were used. Primer pairs 1 and 5 with* BamHI* at 5′ and* XhoI* at 3′ end were used for directional cloning of antitoxin gene (252 bp) in pET28(a). Primer 2 with* Pst*I site at 5′ end and primer 7 with* Hind*III site at 3′ end were used in cloning ORF2 (285 bp) encoding toxin Xn-relE gene in pBAD. Complete TA operon (526 bp) containing antitoxin gene (ORF1) followed by toxin gene (ORF2) was amplified and cloned in pBAD using primer 3 with* Pst*I site at its 5′ end and primer 7 with* Hind*III site at its 3′ end.

Primer pairs used for cloning studies are shown in Figure 1; the sequences details are given in Table 1 and constructs/strains used in this study are listed in Table 2. Primer pairs 4 and 6 with* BamH*I site at 5′ end and* Xho*I site at 3′ end, respectively, were designed against (285 bp) ORF 2 encoding toxin for amplification of Xn-relE gene from the genomic DNA. Amplification product and pGEX4T1 vector were digested with* BamH*I and* Xho*I restriction enzymes and ligated to produce pJSL1 plasmid construct.

Restriction sites BamHI and XhoI were added at 5′ and 3′ end of PCR amplification of antitoxin gene (252 bp) product from genomic DNA using primer sets 1 and 5, respectively, for the directional cloning in pET28(a) vector to produce plasmid pJSL2.

### 2.3. Expression Profile

*E. coli* BL 21(DE3) was transformed with plasmids pJSL1 resulting in strain JSL1 while* E. coli* BL 21(DE3) pLysS cells were transformed with pJSL2 producing JSL2 strain. Both strains were in the expression study of recombinant Xn-relE toxin and Xn-relEAT antitoxin protein. 1 mM IPTG was used to induce transformed strains till culture density reaches OD_600_ = 0.5 for recombinant protein.

### 2.4. Purification

Recombinant Xn-relE was purified with glutathione S-transferase (GST) tag present at the N-terminus using glutathione sepharose affinity chromatography column as per the manufacturer's instructions. Recombinant Xn-relEAT was also purified with Histidine (His) tag at the N-terminus by using Ni-NTA affinity chromatography following the manufacturer's instructions.

Column purified protein was concentrated using Millipore Centricon (PM ~ 10) and dialysed with 100-fold volume of phosphate buffer (50 mM concentration, pH-8) and to avoid the protease activity PMSF was added. After dialysis the protein was retrieved and stored at −20°C in the presence of 15% glycerol.

### 2.5. Cloning in pBAD

For amplification of 285 bp ORF2 encoding toxin Xn-relE gene from the genomic DNA primer 2 with* Pst*I site at 5′ end and primer 7 with* Hind*III site at 3′ end were used. PCR amplified product and expression vector pBAD were double-digested with restriction enzymes* Pst*I and* Hind*III and digestion product was purified using QIAGEN gel extraction kit and ligated to generate pJSL3 plasmid construct.

Complete TA operon (526 bp) containing antitoxin gene (ORF1) followed by toxin gene (ORF2) was amplified using primer 3 with* Pst*I site at its 5′ end and primer 7 with* Hind*III site at its 3′ end. PCR amplified product and pBAD expression vector were digested with* Pst*I and* Hind*III restriction enzymes and ligated producing pJSL4.

### 2.6. Endogenous Toxicity Assay

*E. coli* Top 10 strains were selected for the endogenous toxicity assay and all the constructs of pBAD plasmid were transformed in* E. coli* Top 10 strains resulting in JSL (pBAD), JSL3 (pBAD-Xn-relE), and JSL4 (pBAD-Xn-relE + AT). Overnight grown culture was diluted 100-fold in fresh LB medium for toxicity assay and grown until log phase of growth. When optical density at 600 nm [OD_600_] reaches ~0.4 to 0.5, 0.2% L-(+)-arabinose (Sigma, St. Louis, MO) was added to the culture medium. Cultures growth condition was 37°C in LB medium with 100 mg/mL of Ampicillin and 225 rpm of continuous shaking. All the experiments were performed in triplicate and mean value of their results was used for calculation of growth in percentage (%) at different time intervals.

## 3. Results and Discussion

### 3.1. Cloning, Expression, and Purification

**Figure 2:**
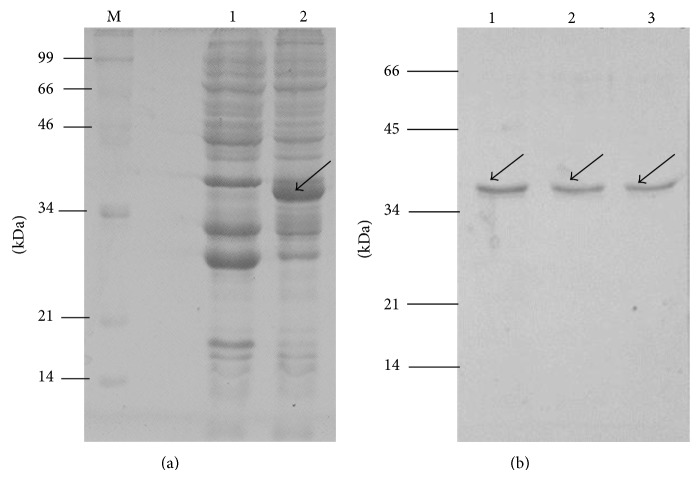
Expression profile of recombinant Xn-relE toxin protein. SDS-PAGE showing expression of Xn-relE toxin gene. (a) Lane M, protein marker; lane 1, induced cells harbouring pGEX4T1 vector; lane 2, induced cells from clone 1. (b) Purification of recombinant GST tagged Xn-relE protein by GST affinity chromatography. Lanes 1, 2, and 3, purified recombinant GST tagged Xn-relE protein.

Toxin-antitoxin modules are mainly categorized in three different classes and class II TA system comprises proteins forming toxin-antitoxin (TA) protein complex [3, 21]. Xn-relE/EAT proteins from* X. nematophila* have shown similarity with proteins from* Rel* family and hence are supposed to form protein complex. Genomic organization of the novel Xn-relE/EAT operon is shown in Figure 1 indicating overlapping 11 base pairs in between. In this study an attempt was made for the expression of Xn-relE gene in pET-28 vector but results were not satisfactory. Then Xn-relE toxin gene alone was cloned in pGEX4T1 expression vector and expression profile is shown in Figure 2(a). Band as shown with arrow in lane 2 was visible in SDS-PAGE at the position above 34 kDa protein marker which is corroborated with the size of GST fusion with Xn-relE recombinant protein. Corresponding band was missing in the induced cells containing empty vector as shown in lane 1 in Figure 2(a). GST tagged recombinant Xn-relE protein was purified with GST affinity chromatography under optimal conditions. Single band was visible in SDS-PAGE at the position below 34 kDa protein marker which is corroborated with the size of GST fusion with Xn-relE recombinant protein as shown in Figure 2(b). From the study it has been confirmed that Xn-relE gene is encoded for a novel toxic protein whose nature and degree of endotoxicity were also studied in this study which signifies its functional similarities with other toxins from Rel family [22–24].

**Figure 3:**
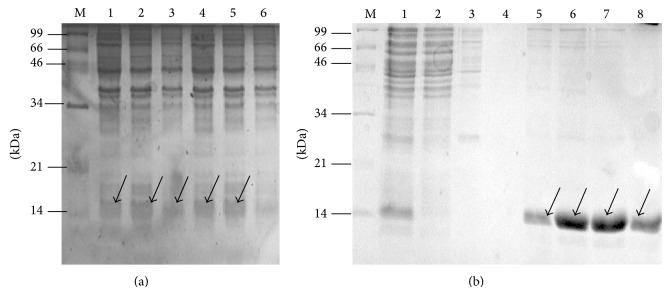
Expression profile of recombinant Xn-relEAT antitoxin protein. SDS-PAGE showing expression of Xn-relEAT protein. (a) Lane M, protein marker; lanes 1, 2, 3, 4, and 5, induced cells from clones 1, 2, 3, 4, and 5; lane 6, induced cells harbouring pET-28 vector. (b) Purification of recombinant His (6x) tagged Xn-relEAT protein by Ni-NTA affinity chromatography. Lane M, protein marker; lane 1, induced cell lysate; lane 2, flow-through; lane 3, wash 1 with 50 mM sodium phosphate buffer; lane 4, wash 2 with 50 mM sodium phosphate buffer containing 20 mM imidazole; lanes 5, 6, 7, and 8, purified recombinant His (6x) tagged Xn-relEAT protein fractions.

*Xn-relEAT* antitoxin gene was also cloned in pET-28 expression vector and expression profile of five clones was determined as shown in Figure 3(a). Bands as shown with arrow in lanes 1, 2, 3, 4, and 5 were visible in SDS-PAGE at the position below 14 kDa protein markers which is corroborated with the size of recombinant His (6x) tagged Xn-relEAT protein. Corresponding band was missing in the induced cells containing empty vector as shown in lane 6 in Figure 3(a). Recombinant His tagged Xn-relEAT protein was purified with Ni-NTA affinity chromatography under native conditions. Single band was visible in lanes 5, 6, 7, and 8 in Figure 3(b) as shown with arrow in SDS-PAGE at the position corresponding to 14 kDa protein marker which is corroborated with the size of recombinant His tagged Xn-RelEAT protein. Hence we also conclude that Xn-relEAT gene from the same operon encodes antitoxin protein which is similar to other RelB antitoxins of Rel family [24].

### 3.2. Endogenous Toxicity Assay

The chromosomal toxin-antitoxin systems perform various cellular functions via cell cycle arrest, stress response mechanisms, and promoting programmed cell death [25, 26].

**Figure 4:**
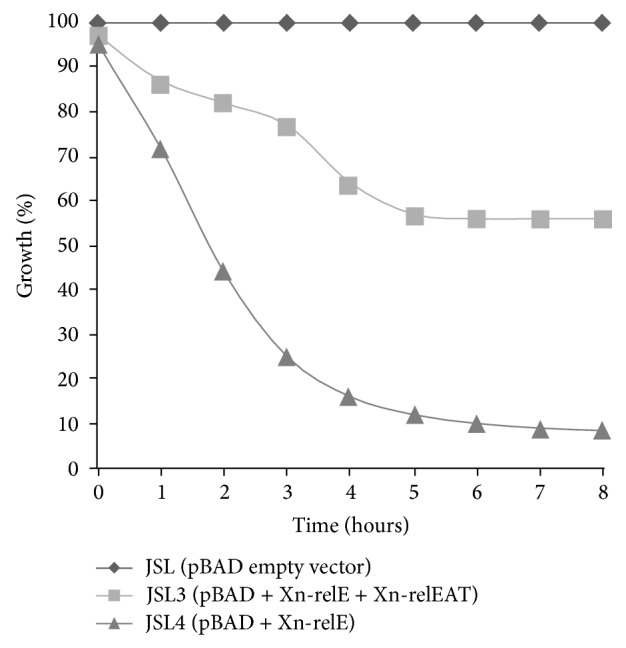
Endogenous toxicity assay. All the experiments were performed in triplicate, and mean values were used to show the results in percentage (%) growth at different time intervals. Bacterial growth was monitored by determining optical density at 600 nm in the presence of arabinose. (◆) JSL strain [WT (wild type) strain + empty vector (control)]; (▲) JSL3 strain [WT (wild type) strain + Xn-relE toxin]; and (■) JSL4 strain [WT (wild type) + operon (Xn-relE + Xn-relEAT)].

Type II toxin acts in a number of ways, but toxin activity is exerted most if it acts as an endonuclease/interferase [27, 28] while antitoxin usually inhibits the toxin by downregulating toxin expression. Under stress condition transcription of TA operon leads to formation of TA complex [10, 14]. Simultaneous expression of Lon protease and its action over labile antitoxin that is susceptible to be degraded ultimately unleash toxin from the toxin-antitoxin complex. Toxins are less susceptible to proteases and their toxic effect is exerted on host cells either by inhibiting cell wall formation or by halting protein synthesis [29]. Therefore, to study the endogenous toxic effect of putative protein from novel identified Xn-relE gene as well as neutralization effect by putative Xn-relEAT gene, both were cloned under tightly regulated ara promoter. pJSL3 recombinant plasmid harbouring Xn-relE gene and pJSL4 recombinant plasmid containing complete TA operon (Xn-relE + Xn-relEAT) were transformed in* E. coli* Top 10 cells. In the endogenous toxic assay, control strain JSL containing pBAD vector alone was considered as 100% based on the growth profile after induction with arabinose when compared with arabinose-induced JSL3 strain containing Xn-relE toxin only as well as with JSL4 strain with full operon (Xn-relE + Xn-relEAT). Results show that there was no change in growth profile of JSL3 strain after the first hour of induction; however, there is gradual decrease in the growth following time intervals. It was inhibited by 25% by the third hour of arabinose induction graph showing steep fall at initial time points and was further declined to 8% after 8 hours in JSL3 strain as compared to control as shown in Figure 4. However, ara induced full length operon was also growth inhibition, but it was less inhibited than that of Xn-relE toxin alone as only ~55% inhibition was observed after eight hours of induction. Reduction in growth inhibition is due to the expression of antitoxin Xn-relEAT from the operon under ara promoter although the expression of antitoxin Xn-relEAT is not able to completely neutralize the toxic effect of Xn-relE gene. Less expression of antitoxin Xn-relEAT which might be incapable of neutralizing the toxicity of Xn-relE makes a clue behind the difference in the growth inhibition profile of arabinose-induced culture. It also indicates that action of toxin is much rapid that it exerts its effects before antitoxin molecule makes a TA complex and neutralizes it. Recent studies showed that free toxins in the cell are very less due to the negative transcriptional control loop as well as tight complex formation between toxin and antitoxin. Moreover, in* E. coli* TA module RelBE they form heterotrimer complex, consisting of two antitoxins (RelB) and one toxin molecule (RelE) bounded together [24, 30]. Therefore, it can be assumed that toxic effect of Xn-relE toxin neutralization may require more than one Xn-relEAT protein molecule. Hence, in endogenous toxicity assay, difference in growth to significant extent was observed between JSL strain [(wild type) strain + empty vector (control)] and JSL4 strain [WT (wild type) + operon (Xn-relE + Xn-relEAT)].

## Figures and Tables

**Table 1:** Primers used in this study.

Primer	Sequence
Primer 1	5′-GGATCC ATG TCT TAT CAG ATC CTG ACA ACA ATA-3′
Primer 2	5′-CTG CAG ATG ACT TAT AGT CTC AAA TTT GAA AAG-3′
Primer 3	5′-CTG CAG ATG TCT TAT CAG ATC CTG ACA ACA-3′
Primer 4	5′-GGATCC ATG ACT TAT AGT CTC AAA TTT GAA AAG-3′
Primer 5	5′-AAGCTT CTA TAA GTC ATT GAG ATC GAC GCT-3′
Primer 6	5′-CTCGAG TTA TTC ACG TTC ATC AGC GAC TGA ATA-3′
Primer 7	5′-AAG CTT TTA TTC ACG TTC ATC AGC GAC TGA-3′

**Table 2:** Strains and plasmid used in this study.

Construct/strain	Characteristic	Source
*E. coli *DH5*α*	*supE44*Δ*lacU169 hsdR17 recA1 endA1 gyrA96 thi-1 relA1*Φ80 *dlacZ *ΔM15	Invitrogen
*E. coli* BL21 (DE3) pLysS	F-*omp*T *hsd*SB(*rB*−*mB*−) *gal dcm*(DE3)plysS(CmR)	Novagen
*E. coli *TOP10	F-Φ80*lac*ZΔM15 Δ(*lac*ZYA-*arg*F) U169 *rec*A1 *end*A1 *hsd*R17 (rK–, mK+) *pho*A *sup*E44 *λ*-*thi*-1 *gyr*A96 *rel*A1	Invitrogen
*E. coli *BL21 (DE3)	F-ompT hsdSB(rB–, mB–) gal dcm (DE3)	Novagen
pET 28 (a)	5.3 kb expression vector; kanr	Novagen
pBAD His (c)	4.1 kb, L-arabinose regulated pBR322-derived expression vectors designed for regulated, recombinant protein expression and purification in *E. coli*.	Invitrogen
pGEX4T1	4.96 kb, bacterial vector for expressing fusion proteins with a thrombin site, GST tagged	GE Healthcare
pJSL/JSL	pBAD His (c) alone without insert in *E. coli *Top 10 cells	Present study
pJSL1/JSL1	pGEX4T vector containing 285 bp RelB toxin gene from RelE TA module of *X. nematophila. *	Present study
pJSL2/JSL2	pET 28 (a) vector containing 252 bp RelE antitoxin gene from RelE TA module of *X. nematophila. *	Present study
pJSL3/JSL3	pBAD vector containing 285 bp RelE toxin gene from RelE TA module of *X. nematophila. *	Present study
pJSL4/JSL4	pBAD vector containing 526 bp full RelE operon from the genome of* X. nematophila. *	Present study

## References

[1] van Melderen L. (2010). Toxin-antitoxin systems: why so many, what for?. *Current Opinion in Microbiology*.

[2] Holčík M., Iyer V. N. (1997). Conditionally lethal genes associated with bacterial plasmids. *Microbiology*.

[3] Gerdes K., Christensen S. K., Løbner-Olesen A. (2005). Prokaryotic toxin-antitoxin stress response loci. *Nature Reviews Microbiology*.

[4] Magnuson R. D. (2007). Hypothetical functions of toxin-antitoxin systems. *Journal of Bacteriology*.

[5] Lewis K. (2010). Persister cells. *Annual Review of Microbiology*.

[6] Christensen S. K., Gerdes K. (2003). RelE toxins from Bacteria and Archaea cleave mRNAs on translating ribosomes, which are rescued by tmRNA. *Molecular Microbiology*.

[7] Pedersen K., Zavialov A. V., Pavlov M. Y., Elf J., Gerdes K., Ehrenberg M. (2003). The bacterial toxin RelE displays codon-specific cleavage of mRNAs in the ribosomal A site. *Cell*.

[8] Donegan N. P., Thompson E. T., Fu Z., Cheung A. L. (2010). Proteolytic regulation of toxin-antitoxin systems by ClpPc in *Staphylococcus aureus*. *Journal of Bacteriology*.

[9] Makarova K. S., Wolf Y. I., Koonin E. V. (2009). Comprehensive comparative-genomic analysis of type 2 toxin-antitoxin systems and related mobile stress response systems in prokaryotes. *Biology Direct*.

[10] Christensen S. K., Mikkelsen M., Pedersen K., Gerdes K. (2001). RelE, a global inhibitor of translation, is activated during nutritional stress. *Proceedings of the National Academy of Sciences of the United States of America*.

[11] Mutschler H., Gebhardt M., Shoeman R. L., Meinhart A. (2011). A novel mechanism of programmed cell death in bacteria by toxin-antitoxin systems corrupts peptidoglycan synthesis. *PLoS Biology*.

[12] de la Hoz A. B., Ayora S., Sitkiewicz I., Fernández S., Pankiewicz R., Alonso J. C., Ceglowski P. (2000). Plasmid copy-number control and better-than-random segregation genes of pSM19035 share a common regulator. *Proceedings of the National Academy of Sciences of the United States of America*.

[13] Hallez R., Geeraerts D., Sterckx Y., Mine N., Loris R., Van Melderen L. (2010). New toxins homologous to ParE belonging to three-component toxin-antitoxin systems in Escherichia coli O157:H7. *Molecular Microbiology*.

[14] Cataudella I., Trusina A., Sneppen K., Gerdes K., Mitarai N. (2012). Conditional cooperativity in toxin-antitoxin regulation prevents random toxin activation and promotes fast translational recovery. *Nucleic Acids Research*.

[15] Leplae R., Geeraerts D., Hallez R., Guglielmini J., Drze P., Van Melderen L. (2011). Diversity of bacterial type II toxin-antitoxin systems: a comprehensive search and functional analysis of novel families. *Nucleic Acids Research*.

[16] Akhurst R. J. (1982). Antibiotic activity of *Xenorhabdus* spp., bacteria symbiotically associated with insect pathogenic nematodes of the families *Heterorhabditidae* and *Steinernematidae*. *Journal of General Microbiology*.

[17] Herbert E. E., Goodrich-Blair H. (2007). Friend and foe: The two faces of *Xenorhabdus nematophila*. *Nature Reviews Microbiology*.

[18] Akhurst R. J., Bedding R., Akhurst R., Kaya H. (1993). Bacterial symbionts of entomopatho-genic nematodes—the power behind the throne. *Nematodes and the Biological Control of Insect Pests*.

[19] Nielsen-LeRoux C., Gaudriault S., Ramarao N., Lereclus D., Givaudan A. (2012). How the insect pathogen bacteria *Bacillus thuringiensis* and *Xenorhabdus/Photorhabdus* occupy their hosts. *Current Opinion in Microbiology*.

[20] Singh J., Chaudhary R. K., Gautam P. (2012). Insilico analysis of novel RelB, RelE and MazF toxin-antitoxin homolog's from the genome of *Xenorhabdus nematophila*. *The American Journal of Bioinformatics Research*.

[21] Yamaguchi Y., Park J.-H., Inouye M. (2011). Toxin-antitoxin systems in bacteria and archaea. *Annual Review of Genetics*.

[22] Cherny I., Overgaard M., Borch J., Bram Y., Gerdes K., Gazit E. (2007). Structural and thermodynamic characterization of the *Escherichia coli* RelBE toxin-antitoxin system: indication for a functional role of differential stability. *Biochemistry*.

[23] Hurley J. M., Cruz J. W., Ouyang M., Woychik N. A. (2011). Bacterial toxin RelE mediates frequent codon-independent mRNA cleavage from the 5' end of coding regions in vivo. *The Journal of Biological Chemistry*.

[24] Griffin M. A., Davis J. H., Strobel S. A. (2013). Bacterial toxin RelE: a highly efficient ribonuclease with exquisite substrate specificity using atypical catalytic residues. *Biochemistry*.

[25] van Melderen L., de Bast M. S. (2009). Bacterial toxin-antitoxin systems: more than selfish entities?. *PLoS Genetics*.

[26] Hayes F. (2003). Toxins-antitoxins: plasmid maintenance, programmed cell death, and cell cycle arrest. *Science*.

[27] Christensen-Dalsgaard M., Overgaard M., Winther K. S., Gerdes K. (2008). RNA decay by messenger RNA interferases. *Methods in Enzymology*.

[28] Yamaguchi Y., Inouye M. (2009). mRNA interferases, sequence-specific endoribonucleases from the toxin-antitoxin systems. *Progress in Molecular Biology and Translational Science*.

[29] Cataudella I., Sneppen K., Gerdes K., Mitarai N. (2013). Conditional cooperativity of toxin—antitoxin regulation can mediate bistability between growth and dormancy. *PLoS Computational Biology*.

[30] Overgaard M., Borch J., Gerdes K. (2009). RelB and RelE of *Escherichia coli* form a tight complex that represses transcription via the ribbon-helix-helix motif in RelB. *Journal of Molecular Biology*.

